# Genotype, Antifungal Susceptibility, and Virulence of Clinical South African *Cryptococcus neoformans* Strains from National Surveillance, 2005–2009

**DOI:** 10.3390/jof7050338

**Published:** 2021-04-27

**Authors:** Serisha D. Naicker, Rindidzani E. Magobo, Tsidiso G. Maphanga, Carolina Firacative, Erika van Schalkwyk, Juan Monroy-Nieto, Jolene Bowers, David M. Engelthaler, Liliwe Shuping, Wieland Meyer, Nelesh P. Govender

**Affiliations:** 1 Center for Healthcare-Associated Infections, Antimicrobial Resistance and Mycoses, National Institute for Communicable Diseases, A Division of the National Health Laboratory Service, Johannesburg 2192, South Africa; rindidzanim@nicd.ac.za (R.E.M.); tsidisom@nicd.ac.za (T.G.M.); e.britz1@gmail.com (E.v.S.); LiliweS@nicd.ac.za (L.S.); Neleshg@nicd.ac.za (N.P.G.); 2 School of Pathology, Faculty of Health Sciences, University of the Witwatersrand, Johannesburg 2001, South Africa; 3 Studies in Translational Microbiology and Emerging Diseases (MICROS) Research Group, School of Medicine and Health Sciences, Universidad del Rosario, 111611 Bogota, Colombia; cfiracative@gmail.com; 4 Pathogen and Microbiome Division, Translational Genomics Research Institute, Phoenix, AZ 85004, USA; jmonroy-nieto@tgen.org (J.M.-N.); jbowers@tgen.org (J.B.); dengelthaler@tgen.org (D.M.E.); 5 Molecular Mycology Research Laboratory, Center for Infectious Diseases and Microbiology, Westmead Clinical School, Sydney Medical School, Faculty of Medicine and Health, The University of Sydney, Sydney, NSW 2006, Australia; wieland.meyer@sydney.edu.au; 6 Marie Bashir Institute for Emerging Infectious Diseases and Biosecurity, University of Sydney, Sydney, NSW 2006, Australia; 7 Westmead Institute for Medical Research, Westmead, NSW 2145, Australia; 8 Research and Educational Network, Westmead Hospital, Western Sydney Local Health District, Westmead, NSW 2145, Australia; 9 Division of Medical Microbiology, Faculty of Health Sciences, University of Cape Town, Cape Town 7701, South Africa

**Keywords:** *Cryptococcus neoformans*, molecular type, South Africa, MLST, whole genome sequencing, fluconazole, antifungal resistance

## Abstract

In South Africa, *Cryptococcus neoformans* is the most common cause of adult meningitis. We performed multi locus sequence typing and fluconazole susceptibility testing of clinical *C. neoformans* isolates collected from 251 South African patients with cryptococcosis through national surveillance from 2005 to 2009. We examined the association between clinical characteristics of patients and genotype, and the effect of genotype on in-hospital mortality. We performed whole genome phylogenetic analysis of fifteen *C. neoformans* isolates with the molecular type VNB and tested their virulence in a *Galleria mellonella* model. Most isolates had the molecular type VNI (206/251, 82%), followed by VNII (25/251, 10%), VNB (15/251, 6%), and VNIV (5/251, 2%); 67 sequence types were identified. There were no differences in fluconazole minimum inhibitory concentration (MIC) values among molecular types and the majority of strains had low MIC values (MIC_50_ of 1 µg/mL and MIC_90_ of 4 µg/mL). Males were almost twice as likely of being infected with a non-VNI genotype (adjusted odds ratio [OR]: 1.65, 95% confidence interval [CI]: 0.25–10.99; *p* = 0.61). Compared to patients infected with a VNI genotype, those with a non-VNI genotype had a 50% reduced adjusted odds of dying in hospital (95% CI: 0.03–7.57; *p* = 0.62). However, for both these analyses, our estimates had wide confidence intervals spanning 1 with large *p*-values. Fifteen VNB strains were not as virulent in a *G. mellonella* larval model as the H99 reference strain. A majority of these VNB strains belonged to the VNBII clade and were very closely related by phylogenetic analysis.

## 1. Introduction

*Cryptococcus* is a more common cause of meningitis among adults in South Africa than bacteria or *Mycobacterium tuberculosis*, accounting for 62% of microbiologically-confirmed cases [1]. Two species-complexes within the genus *Cryptococcus* cause most human disease: *Cryptococcus neoformans* and *Cryptococcus gattii* [2]. Both are known to cause disease in HIV-infected persons and immunocompetent individuals [2]. The ecological niche of these sibling species overlaps in southern Africa including soil, bird droppings, and certain tree species [3]. For induction treatment of HIV-associated cryptococcal meningitis, flucytosine is now recommended in combination with amphotericin B, followed by fluconazole monotherapy for consolidation and maintenance treatment [4].

Multi locus sequence typing (MLST) is a good discriminatory tool to genotype strains of the two closely-related sibling *Cryptococcus* species and is the gold standard for identifying the major molecular types of *C. neoformans* and *C. gattii* [5]. Molecular typing subdivides *C. neoformans* into five molecular types: VNI, VNB, VNII, VNIII, and VNIV [6]. New species names that correspond to each molecular type were proposed due to differences in virulence, geographic range, epidemiology, and population genetics [7]. While this proposal may not be formalized because some members of the scientific community foresaw issues of potential taxonomic instability, others have adopted this nomenclature in the published literature [8]. A comprehensive review of published *C. neoformans* genotyping studies from South America (1766 isolates), Asia (1444 isolates), Europe (1217 isolates), North America (938 isolates), Africa (475 isolates), and Oceania (189 isolates) summarized the molecular type distribution of *C. neoformans* on a global scale [9]. VNI is distributed worldwide and is the most prevalent molecular type [9]. VNB is predominately found in sub-Saharan Africa (clinical and environmental isolates), but recently-sequenced environmental and clinical isolates from Brazil had this genotype [10,11]. VNII and VNIII are less frequently found globally [9]. VNIV also has global distribution but is more frequently found in Europe [9].

African environmental isolates of VNI and VNII are associated with urban areas, pigeon excreta, and non-Mopane trees whereas VNB is mostly associated with *Colophospermum mopane* (Mopane) trees [12,13,14]. Studies done in Zambia and Botswana further suggest that VNI infections are associated with urbanized populations while VNB is associated with Mopane trees in rural settings [13,14]. Whole genome sequencing (WGS) has revealed that VNB isolates have a higher diversity compared to VNI and VNII isolates [15]. Two groups have proposed that the VNB lineage be split into two clades, namely VNB-A and VNB-B [14] or VNBI and VNBII [16]. Population genomic analyses of VNB isolates showed that the VNB lineage migrated bi-directionally between Africa and South America before its diversification into these two clades [15]. Additionally, patients infected with VNB in a South African study had a significantly worse survival outcome, suggesting that the genetic lineage plays an integral role during infection and that there is possibly a link between clinical characteristics and genotype [17].

We aimed to describe the molecular type distribution and fluconazole susceptibility of clinical South African *C. neoformans* isolates collected through national laboratory-based surveillance during 2005–2009. We examined the association between clinical characteristics of patients and molecular type, and the effect of molecular type on in-hospital mortality. We also sequenced the genomes of fifteen VNB isolates from our study and compared the virulence of these strains to the H99 reference strain in a *Galleria mellonella* model. We focused our Whole Genome Sequencing (WGS) and virulence studies on this molecular type since this genotype is endemic to southern Africa and based on previous work, hypothesized that this genotype may be more virulent than the other molecular types [17].

## 2. Materials and Methods

### 2.1. Study Design and Sample Selection

We conducted a cross-sectional study nested within national laboratory-based surveillance for cryptococcosis in South Africa from 2005 through to 2009 [18]. A case was defined as a person diagnosed with cryptococcal disease by any one of the following positive laboratory tests during a 30-day period: India ink microscopy on cerebrospinal fluid (CSF), cryptococcal antigen test on blood or CSF, and/or culture of *Cryptococcus* from any specimen. At least 50 *C. neoformans* isolates from each of the surveillance years, 2005 to 2009, were randomly selected for genotyping using a random-integer generator (https://www.random.org/integers/, accessed on 4 January 2010), totaling 251 cases. In some years, more than 50 isolates were selected to account for non-viability and/or contamination. Recurrent isolates from the same patients were excluded and in such instances, the incident isolate was the one selected. We also excluded isolates that were identified as *C. gattii* or other *Cryptococcus* species. We genotyped 251 strains from a total of 18,040 cases between 2005 and 2009. We then estimated the prevalence and 95% CI for each genotype in this random sample of the study population. We applied the calculated 95% CIs to the study population of 18,040 cases to determine the range of cases per genotype in the study population.

### 2.2. Subculture and Identification of Surveillance Isolates

Following primary isolation in culture at diagnostic laboratories, a sweep of the culture plate was inoculated onto Dorset medium (Diagnostic Media Products (DMP), NHLS) and transported to a reference laboratory at the National Institute for Communicable Diseases (NICD) in Johannesburg, where the isolates were identified and stored at −70 °C. For this study, the *C. neoformans* isolates were retrieved from −70 °C storage and sub-cultured onto Sabouraud dextrose agar (DMP) to check for purity and viability. We characterized isolates phenotypically as previously described [5].

### 2.3. Multi Locus Sequence typing (MLST) Experiments and Data Analysis

DNA was extracted from single yeast colonies using the Zymo ZR Fungal/Bacterial DNA MiniPrep Kit (Zymo Research Corp, Irvine, CA, USA) following the manufacturer’s instructions. Thereafter, DNA amplification and sequencing was performed for six housekeeping genes *CAP59*, *GPD1*, *LAC1*, *PLB1*, *SOD1*, *URA5*, and the intergenic spacer region *IGS1*, as described by Meyer et al. [6]. For each locus, DNA sequences in both forward and reverse orientations were obtained and edited using Sequencher 5.4.6. Allele numbers and sequence types (STs) were assigned using the MLST database (http://mlst.mycologylab.org). Concatenated DNA sequences for the seven loci were aligned using the program ClustalW (BioEdit Sequence Alignment Editor). A phylogenetic tree was generated in the program MEGA using the neighbor-joining method with a bootstrap analysis of 100 replicates and the Jukes–Cantor model. With the number and frequency of STs, the Simpsons diversity index (*D*) was calculated to estimate the genetic diversity of *C. neoformans* strains from this study. High scores (close to 1) indicate high diversity and low scores (close to 0) indicate low diversity [19]. Mating type identification was performed by polymerase chain reaction (PCR) amplification using MFα primers (upper and lower sets) and the STE20_SF_a primer sets, which are specific to the mating type regions of α and a mating-type cells, respectively, according to a previous study [20].

### 2.4. Fluconazole Susceptibility Testing

Of the 251 *C. neoformans* isolates, antifungal susceptibility testing was performed for 105 isolates: 60 randomly-selected VNI (from a total of 206), 25 VNII, 15 VNB, and five VNIV. We determined fluconazole minimum inhibitory concentration (MIC) values (range: 0.125 µg/mL to 64 µg/mL) using custom-made broth microdilution panels (NICD, Johannesburg) prepared, inoculated, and read according to susceptibility testing methods published previously and Clinical and Laboratory Standards Institute M27-A3 and M60 recommendations [21,22,23]. We interpreted fluconazole MIC values using published epidemiological cutoff values (ECVs) [24].

### 2.5. Virulence Studies

To assess the virulence of the 15 VNB strains in our study, we used the moth *G. mellonella* model of fungal disease [25]. Larvae in the final instar stage of larval development were used in experiments according to methods published previously [26,27]. Briefly, ten larvae per VNB strain were injected with 10 µL of a 10^8^ yeast cells/ml inoculum prepared using normal saline. Ten larvae were also inoculated with a highly-virulent reference strain (*C. neoformans* H99). We also included two sets of control groups, whereby we injected 10 larvae each with phosphate-buffered saline (PBS) and another 10 larvae each that were not injected. Following injection, larvae were incubated in Petri dishes at 37 °C in the dark and the number of dead larvae were recorded daily for ten days. Larvae were considered dead when they did not move in response to a physical stimulus such as a sterile forceps. We performed three experimental replicates and an average from the three replicates was calculated. We generated a Kaplan–Meier survival curve for larvae infected with each VNB strain, the reference strain H99, and the controls. As a proxy for virulence, we compared the median survival times of larvae infected with each VNB strain to larvae infected with the well-studied reference strain H99 (genotype VNI). Details of the statistical analysis are below.

### 2.6. Whole Genome Sequencing and Phylogenetic Analysis

Whole genome sequencing was performed on 15 isolates with the molecular type VNB using Illumina MiSeq sequencing technology (Illumina, San Diego, CA, USA). DNA samples were prepared for paired-end sequencing using the NEBNext Ultra II DNA Library Prep Kit for Illumina followed by 2 × 300 bp sequencing on a MiSeq instrument. FASTQC and PrinSeq were used to check the quality of read data and perform read filtering. Sequenced genomes were then aligned to a known reference VNB genome (Bt 85, NCBI BioSample number: SAMN01162765) to compare and call single nucleotide polymorphism (SNP) variants using Burrows-Wheeler Aligner (BWA) and SAMtools. BWA and SAMtools were performed using the publicly-available Northern Arizona SNP Pipeline (NASP) [28]; filtering parameters involved removing positions that had <10x coverage, <90% variant allele calls, and those within duplicated regions in the reference. Downstream filtering by NASP produced the final BestSNP alignment that was then used for maximum parsimony inference. Only positions present in all genomes with at least 10x depth of coverage and 90% agreement were included. Maximum parsimony trees with 500 bootstrap replicates were constructed and visualized using MEGA software. In our first analysis, we included whole genome sequences of publicly-available VNB strains from the study conducted by Rhodes et al. in 2017; this included 10 strains from Botswana, seven from South Africa, and four from Brazil [15]. From this initial analysis, we were able to calculate the distances of samples most closely related to our 15 VNB strains using MinHash. This allowed us to determine a cut-off distance *p*-value of 0.0157. There were 95 publicly-available VNBII isolates that were equal to or less than this value, which we included in our next analysis. These strains were from Botswana (*n* = 56), South Africa (*n* = 20), Zambia (*n* = 16), and Brazil (*n* = 3) [15,16]. In this analysis, we used a draft assembly of one of the 15 VNB strains from our study (isolate number 1239) as a reference using BWA and SAMtools in NASP in order to find publicly-available VNB genomes closely related to our strains.

### 2.7. Statistical Analyses

We used logistic regression models to determine associations between patient characteristics and genotype. Patient characteristics were obtained by nurse surveillance officers at the surveillance sites who either interviewed patients or reviewed patients’ medical charts to obtain detailed case information. Mental status was categorized as “Alert” (Glasgow Coma Scale [GCS] score of 15) or “Not alert” (GCS score < 15 or recorded as disorientated, stuporose, or comatose). We defined genotype as VNI vs. non-VNI (including VNII and VNB), since there were too many sequence types with low frequencies. We therefore compared cases of *C. neoformans* serotype A infection (which corresponds to molecular types VNI, VNII, and VNB) in this analysis and excluded *C. neoformans* serotype D (corresponds to molecular type VNIV). We included the following variables in the final regression model with genotype as the dependent variable: sex, age, year of diagnosis, province, specimen type, mental status at diagnosis, CD4+ T-cell (CD4) count at diagnosis, antiretroviral treatment, CSF India ink result, CSF white cell count, current antifungal treatment, and tuberculosis treatment. This was an exploratory analysis whereby we adjusted for age (added a priori), mental status, CD4 count at diagnosis, and CSF white cell count since these variables have been associated with molecular type in previous studies [17,29]. We also modeled the effect of molecular type on in-hospital mortality, adjusting for sex, age, mental status at diagnosis, CD4 count at diagnosis, antiretroviral treatment, and current antifungal treatment. We also compared the fluconazole MIC_50_ values of the VNI isolates and non-VNI isolates using a Wilcoxon rank-sum test. We performed a log rank test of equality to compare survival curves of larvae infected with H99 and the 15 VNB isolates, where the null hypothesis was that there was no difference between the population survival curves. Statistical analyses were performed using Stata statistical software (Version 14.1; StataCorp LP, College Station, TX, USA).

### 2.8. Ethics Approval

Ethics clearance for this study was obtained from the Human Research Ethics Committee (Medical), University of the Witwatersrand. We obtained an ethics waiver from the Animal Ethics Research Committee, University of the Witwatersrand (Registration number: AREC-101210-002) for the *G. mellonella* virulence studies.

## 3. Results

### 3.1. Descriptive Analysis of National Laboratory-Based Surveillance

From 2005 to 2009, 18,756 viable *Cryptococcus* isolates were collected from 18,534 cases of cryptococcal disease. Ninety-seven per cent of viable isolates (18,260/18,756) were identified as *C. neoformans* originating from 18,040 patients. Due to changes in surveillance methodology, the proportion of culture-confirmed *C. neoformans* cases by year was: 2005: 22% (3933/18,040), 2006: 26% (4686/18,040), 2007: 26% (4726/18,040), 2008: 15% (2768/18,040), and 2009: 11% (1927/18,040). The difference in the proportion of culture-confirmed cases was due to the number of surveillance sites decreasing in 2008–2009. The median age of patients was 34 years (inter-quartile range [IQR]: 29–41 years) and 53% (9616/18,040) were female. Of the 18,040 cases, most patients were diagnosed in Gauteng Province (33%, 5971), followed by KwaZulu-Natal Province (23%, 4194), Mpumalanga Province (9%, 1547), Eastern Cape Province (9%, 1639), North West Province (9%, 1554), and Western Cape Province (7%, 1282). The rest were diagnosed in the Free State (6%, 1051), Limpopo (3%, 599), and Northern Cape (1%, 203) provinces. CSF was the most common specimen type (17,288/18,040, 96%), followed by blood culture (715/18,040, 4%).

### 3.2. Genotyping of 251 Clinical C. neoformans Isolates

**Figure 1:**
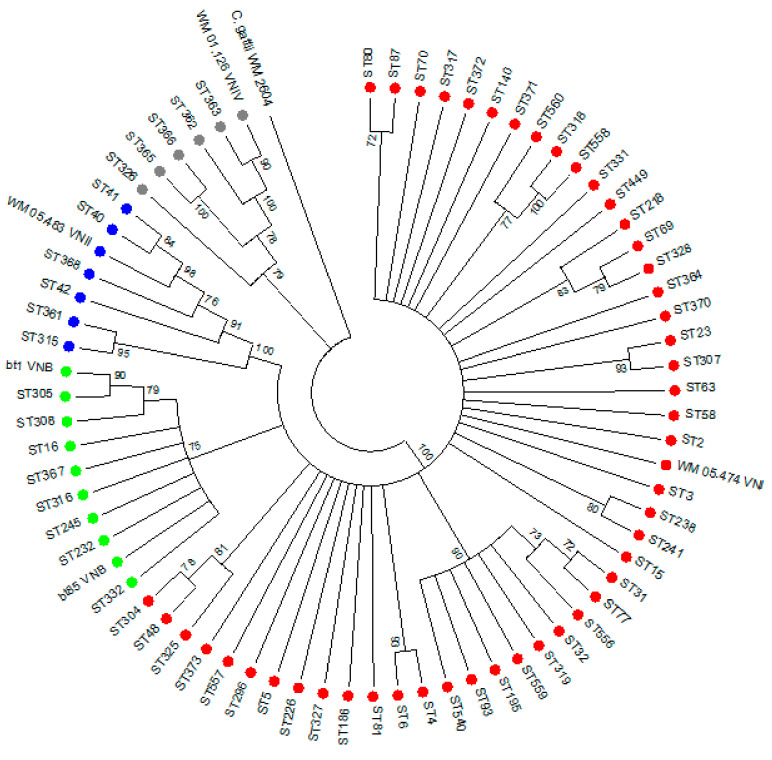
Neighbor joining phylogenetic tree showing clustering of molecular types for 67 different sequence types (STs) from 251 South African clinical *Cryptococcus neoformans* isolates. *Cryptococcus gattii* WM 2604 serves as the outgroup. Red circles indicate the STs with the VNI molecular type, blue circles indicate the STs with the VNII molecular type, green circles indicate the STs with the VNB molecular type, and grey circles indicate the STs with the VNIV molecular type. Bootstrap values are shown next to the branches.

We genotyped 251 strains from a total of 18,040 cases between 2005 and 2009, which equaled 1.4% of all cases. The molecular type distribution of 251 *C. neoformans* isolates (see Supplementary Materials for metadata) were as follows: VNI (206/251, 82%; 95% CI: 0.77–0.87), VNII (25/251, 10%; 95% CI: 0.07–0.14), VNB (15/251, 6%; 95% CI: 0.03–0.10), and VNIV (5/251, 2%; 95% CI: 0.01–0.05). The characteristics of the five cases infected with the VNIV strains are described in Table S1. Most isolates had the mat-α mating type (*n* = 249, 99%) whereas 1% (*n* = 2 VNI isolates) were mat-a. There were 67 different STs observed from 251 *C. neoformans* isolates and these are shown in Figure 1. ST5 (53/251, 21%), ST93 (30/251, 12%), and ST23 (26/251, 10%) were the most commonly-observed types. The genetic diversity of South African *C. neoformans* isolates from this study was high, as determined by calculating the Simpsons diversity index (*D* = 0.9205). We calculated that among the 18,040 cases, VNI would have accounted for 13,891–15,695 cases, VNII for 1263–2526 cases, VNB for 541–1804 cases, and VNIV for 180–902 cases.

### 3.3. Association between Patients’ Clinical Characteristics and Genotype

On multivariable analysis, males were almost twice as likely to be infected with a non-VNI genotype compared to females, after adjusting for age, mental status at diagnosis, CD4 count at diagnosis, and CSF white cell count. Although the point estimate for the odds ratio was 1.65, the 95% CI spanned 1 (0.25–10.99) and the *p*-value was large (0.61) (Tables S2 and S3). This also makes our findings consistent with males having a 75% reduced odds to an eleven-fold increased odds of being infected with non-VNI (95% CI: 0.25–10.99; *p* = 0.61).

### 3.4. Association between Genotype and In-Hospital Mortality

The overall crude in-hospital case fatality ratio was 36% (64/180). The crude case fatality ratio among patients infected with each molecular type was as follows: VNI 37% (54/145), VNII 35% (7/20), VNB 18% (2/11), and VNIV 25% (1/4). Patients infected with a non-VNI cryptococcal molecular type had a case fatality ratio of 29% (9/31) versus 37% (54/145) for patients infected with a VNI genotype (crude odds ratio [OR]: 0.69, 95%CI: 0.29–1.61, *p* = 0.39) (Table S4). Compared to patients infected with a VNI genotype, those with a non-VNI genotype had a 50% reduced odds of dying after adjusting for sex, age, mental status at diagnosis, CD4 count at diagnosis, antiretroviral treatment, and current antifungal treatment. Although the point estimate for the odds ratio was 0.50, the 95% CI spanned 1 (0.03–7.57) and the *p*-value was large (0.62) (Table S5).

### 3.5. Fluconazole Susceptibility Testing

The fluconazole MIC_50_ and MIC_90_ values for 105 *C. neoformans* isolates was 1 µg/mL and 4 µg/mL, respectively, with a geometric mean of 1.32 µg/mL (Table 1). The MIC_50_ value for five VNIV isolates was 2 µg/mL compared to 1 µg/mL for 60 VNI isolates, 25 VNII isolates, and 15 VNB isolates, respectively (Table 1). There was no difference in the MIC_50_ values between the VNI isolates and non-VNI isolates (*p* = 0.17). Most isolates had a MIC value of less than or equal to 8 µg/mL, which is considered to be wild-type according to published ECVs [24]. There was only one VNI isolate that had a MIC of 32 µg/mL.

### 3.6. Virulence and Whole Genome Sequencing (WGS) of 15 C. neoformans Isolates with the VNB Molecular Type

**Figure 2:**
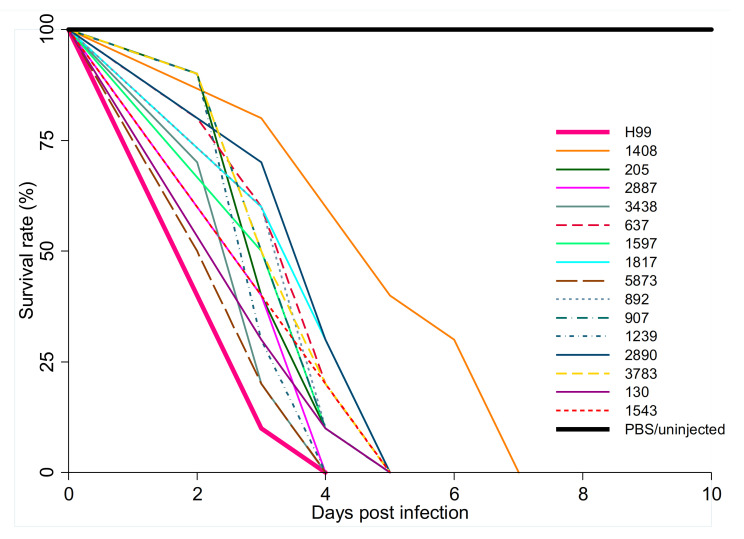
Survival curves of *Galleria mellonella* larvae (30 larvae per strain from three experimental replicates) infected with 15 clinical South African *Cryptococcus neoformans* molecular type VNB isolates. Larvae (*n* = 10 each) were also inoculated with H99 (pink solid line), which is a highly virulent reference strain, or phosphate buffered saline (PBS) or not injected (black solid line).

We plotted survival curves for *G. mellonella* larvae inoculated with 15 *C. neoformans* VNB isolates and H99 (Figure 2). Median survival times and *p*-values are shown in Table 2. The 15 VNB strains showed some heterogeneity in their virulence, as some strains killed as quickly as H99, while others killed the larvae more slowly. The larvae inoculated with one VNB strain (isolate number 5873) had a survival time of two days, which was the same as shown for the highly virulent reference strain H99. Nine VNB strains resulted in a median survival time of the inoculated larvae of three days, four VNB strains resulted in a median survival time of the inoculated larvae of four days, and one strain in a median survival time of the inoculated larvae of five days (Figure 2).

**Figure 3:**
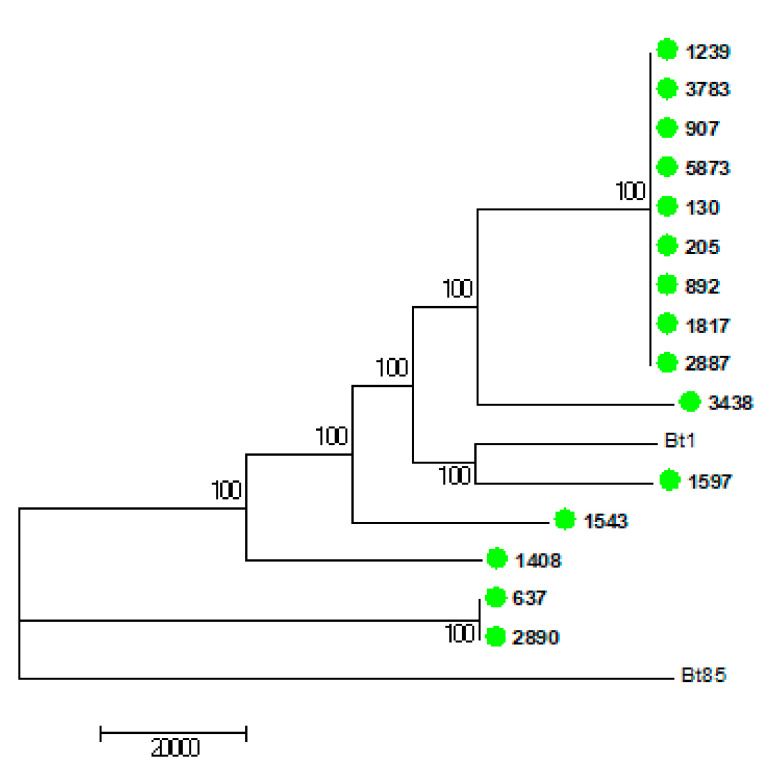
Whole genome sequencing single nucleotide polymorphism (WGS SNP) analysis of 15 South African clinical *Cryptococcus neoformans* isolates with the VNB molecular type (green circles and bold). Bt 85 (VNBI) was used as the reference and Bt 1 (VNBII) was added as an external genome. BWA was used to align reads to the reference and SAMtools was used to call SNPs. There were 293,484 SNPs in total with a coverage breadth of 88% and this is a midpoint-rooted maximum parsimony tree with 500 bootstrap replicates. The bootstrap values are shown next to the branches, the consistency index was 0.65, and the number of phylogenetic sites was 293,484.

**Figure 4:**
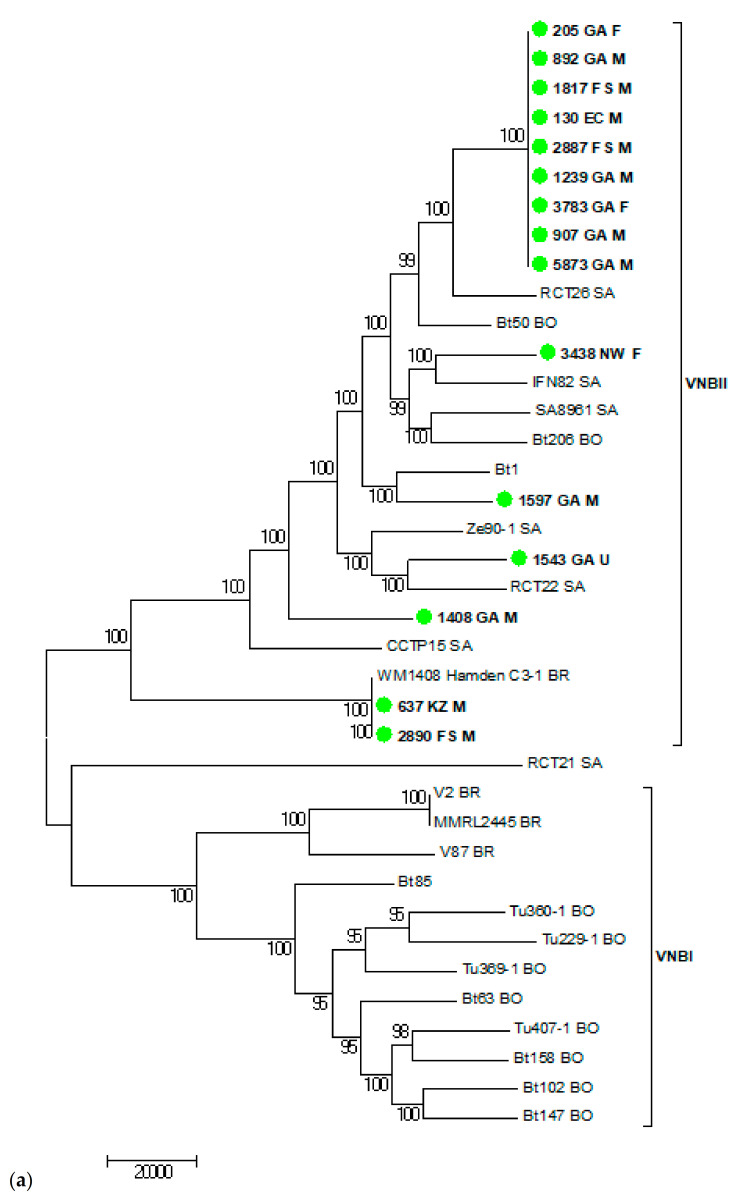

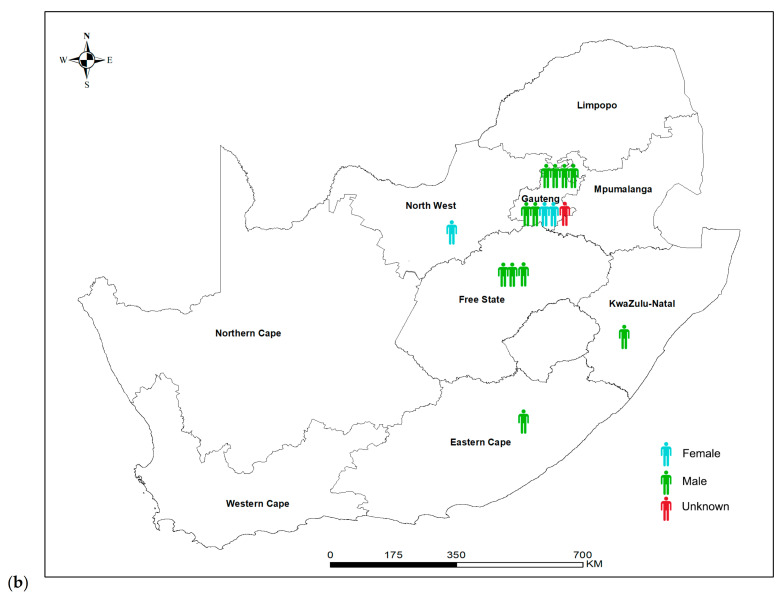
(**a**) WGS SNP analysis of 15 South African clinical *Cryptococcus neoformans* isolates with the VNB molecular type (green circles and bold) and 21 publicly-available clinical and environmental VNB isolates [15]. Bt 85 (VNBI) was used as the reference and Bt 1 (VNBII) was added as an external genome. BWA was used to align reads to the reference and SAMtools was used to call SNPs. There were 488,188 SNPs in total with a coverage breadth of 79% and this is a midpoint-rooted maximum parsimony tree with 500 bootstrap replicates. The bootstrap values are shown next to the branches, the consistency index was 0.39, and the number of phylogenetic sites was 488,188. GA—Gauteng, FS—Free State, EC—Eastern Cape, NW—North West, KZ—KwaZulu-Natal, M—Male, F—Female, U—Unknown, SA—South Africa, BO—Botswana, BR—Brazil. (**b**) The map shows the geographical and gender distribution of the 15 South African patients infected with *C. neoformans* molecular type VNB in our study.

**Figure 5:**
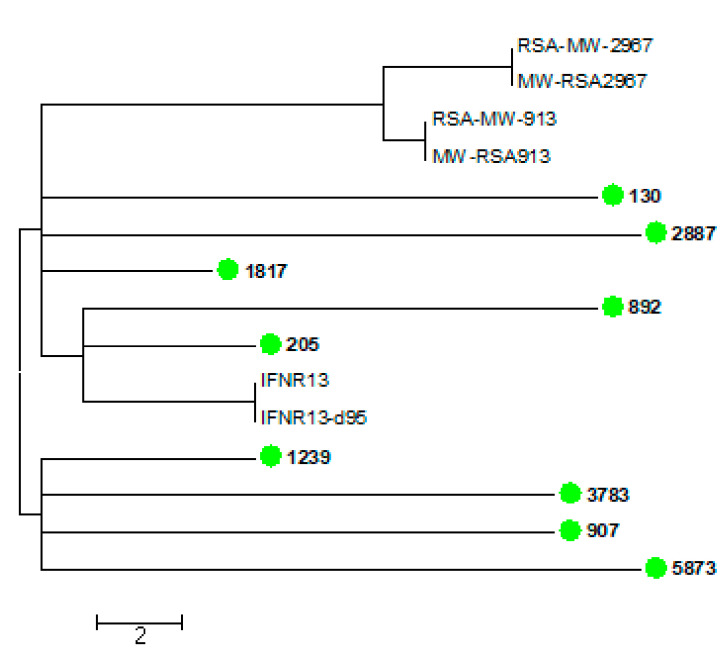
WGS SNP analysis of 15 South African clinical *Cryptococcus neoformans* isolates (nine from this study and six from previous South African studies) with the VNB molecular type. Isolates from this study are indicated with green circles and bold. Sample number 1239 was used as the reference for the VNBII clade. BWA was used to align reads to the reference and SAMtools was used to call SNPs. There were 111 SNPs in total with a coverage breadth of 73% and this is an unrooted maximum parsimony tree. The consistency index was 1.00 and the number of phylogenetic sites was 111.

The WGS results of the 15 VNB isolates are shown in Figure 3, Figure 4 and Figure 5. Figure 3 shows the 15 VNBs including two reference strains for the VNBI and VNBII clades. All 15 VNB isolates clustered in the VNBII clade. Figure 4 shows the 15 VNBs and 21 publicly-available clinical and environmental samples obtained from the study conducted by Rhodes and colleagues [15] with reference strains for the two representative clades. The cluster clade at the top of the tree in Figure 4 consists of nine of our VNB strains with fewer than 35 SNPs among them. Two patients from whom these strains were cultured were female and seven were male. Six patients were from Gauteng Province and had been diagnosed at the same health care facility, another two were from the Free State Province, and one from the Eastern Cape Province. The cases did not cluster in time: two patients were diagnosed in January and August of 2005, four patients in February, April, and November of 2006, and one patient each in March 2007, October 2008, and January 2009, respectively (Table S6). Four other isolates were scattered throughout the tree within the VNBII clade. Two patients were male, one patient was female, and the gender was unknown for one patient. Three patients were from Gauteng Province and one patient was from the North West Province. The remaining two VNBs (sample numbers 637 and 2890) clustered closely with an environmental Brazilian isolate (WM1408 Hamden C3-1 BR); there were fewer than 160 SNPs among the three isolates, but fewer than 30 SNPs between the two South African VNBs. Both patients were male and one was from the Free State Province and one from KwaZulu-Natal Province.

We then performed a comprehensive analysis consisting of 95 publicly-available VNBII isolates from Botswana, South Africa, and Brazil. Six South African strains isolated from patients with recurrent cryptococcosis from previous studies [30,31] were found to be closely related to nine of our strains within the cluster clade with fewer than 12 SNPs between them. Figure 5 shows the WGS SNP analysis using BWA and SAMtools that zoomed in on these 15 closely-related VNB strains (nine from this study and six from previous South African studies). There were fewer than 30 SNP differences among these strains, further confirming that these strains are very closely related. In the comprehensive analysis, we also observed that two of our isolates were closely related to two Brazilian isolates (one being environmental and one being clinical) with fewer than 48 SNPs among the four isolates, while there were fewer than nine SNPs between our two VNB strains.

## 4. Discussion

In summary, most of the 251 South African clinical *C. neoformans* strains in our study had the VNI molecular type, followed by VNII, VNB, and VNIV. There were no differences in fluconazole MIC values among molecular types and the majority of strains had low MIC values within the wild-type range. Males were more likely to be infected with a non-VNI genotype and patients infected with the non-VNI genotype were less likely to die in hospital, though for both of these analyses, our estimates had wide confidence intervals spanning 1 with large *p*-values. There was some heterogeneity in virulence between 15 VNB isolates in a *G. mellonella* larval model compared to the highly-virulent H99 strain. WGS data analysis showed that the 15 VNBs isolated in our study were very closely related and belonged to the VNBII clade.

VNI was the major molecular type identified in our study, which is consistent with the global molecular type distribution [9]. In Africa, VNI accounts for the highest percentage, followed by VNB and VNII, and a small percentage of VNIII [9]; this has been further confirmed by recent molecular epidemiology studies from Cameroon [32], Kenya [33], Ivory Coast [34], and Zimbabwe [35]. VNIV is mostly found in Europe and we found a few isolates with this molecular type in our study. VNIV has also been found in low percentages in Oceania, Asia, and in the Americas [9].

We observed 67 genotypes among these clinical South African *C. neoformans* strains, suggesting a high genetic diversity (*D* = 0.9205). There were 65 unique genotypes in a southern African study, of which 26 genotypes have only been found in this region previously [12]. MLST analysis of *C. neoformans* isolates from pediatric patients from the same South African surveillance system used in our study showed 24 distinct sequence types (ST) [36]. Genotyping analysis of global *C. neoformans* var. *grubii* molecular type VNI isolates by MLST revealed that the highest genetic diversity was observed in African isolates [37]. Beale and colleagues analyzed 230 *C. neoformans* isolates from HIV-seropositive South African clinical trial patients and obtained 50 different STs [17]. Due to the high genetic diversity of African *C. neoformans* clinical and environmental strains, it has been hypothesized that this organism originated in Africa and then spread globally to the rest of the world. Litvintseva et al. termed this the “Out of Africa” hypothesis [12].

The *C. neoformans* isolates in this study, collected between 2005 and 2009, had fluconazole MIC_50_ and MIC_90_ values of 1 µg/mL and 4 µg/mL, respectively, and were therefore considered wild-type. We have recently shown that fluconazole MIC values of isolates from first episodes of meningitis increased between 2007–2008 and 2017 [23], providing evidence for higher fluconazole dose recommendations in the 2019 Southern African guideline for HIV-associated cryptococcosis [4]. In our current study, we found no association between MIC value and genotype. A Zimbabwean study found similar results [35]; whereas other studies have found that VNI isolates had a higher geometric mean fluconazole MIC compared to VNII [38] and VNIV isolates had a lower fluconazole MIC than VNI and VNIII isolates [39].

Previous studies have shown an association between HIV status [40], clinical outcome [10,17,29], altered mental status [17], host immune response [29], and genotype. More specifically, patients infected with the VNB genotype had a worse survival compared to those patients infected with VNI [17], suggesting that this molecular type is more virulent. However, in our study, we could not conclude that our 15 VNB strains were more virulent than H99 in a *G. mellonella* larval model. We only found one strain with a median larval survival time to H99. Furthermore, we observed heterogeneity in virulence between the 15 VNB strains. While we observed that patients infected with the non-VNI genotype (VNII and VNB) were less likely to die compared to patients infected with the VNI genotype, our sample size for patients infected with the non-VNI genotype was small and the 95% CI crossed 1 with a large *p*-value. In a previous study, patients infected with ST93 and ST77 isolates had a 79% mortality when compared to patients infected with ST5 and ST63 within the VNI genotype [29]. Miglia and colleagues found an unadjusted association between gender and ST in a South African pediatric population (84% of males compared to 16% of females, *p* = 0.01). This finding was subject to confounding due to lack of adjusting [36]. We found that males were more likely to be infected with a non-VNI genotype on adjusted analysis, but again, the 95%CI for this effect spanned 1 with a large *p*-value.

Phylogenetic analysis showed that nine of our VNB isolates clustered closely together, suggesting a common environmental source. However, these nine patients resided in the Gauteng, Free State, and Eastern Cape Provinces and therefore spread across different parts of South Africa, thus making it difficult to draw a firm conclusion on how these strains may be related or may have originated. Six South African strains isolated between 2005 and 2010 from patients with recurrent infection from two previous studies [30,31] were very closely related to our nine strains, suggesting a possible outbreak of *Cryptococcus*. Alternatively, it is also possible that the isolates were related by chance considering that there was an estimated total of between 541 and 1804 VNB isolates in 2005–2009.

Two of our VNB strains were more closely related to two Brazilian isolates than to the other South African isolates in our study, suggesting an association between the two South African strains and the two Brazilian strains. There may be more similarity between geographically-distinct VNB isolates and as more VNB isolates are identified elsewhere, especially from South America, we can study this further. Genomic evidence suggests that there were ancient migrations of the VNB molecular type bi-directionally between Africa and South America, and that South American VNB isolates are highly diverse [15]. In addition, VNB isolates from South America have experienced ancestral recombination and donated genetic material to all molecular types across different geographical areas [15].

In conclusion, our study shows that South African clinical *C. neoformans* isolates collected through national laboratory-based surveillance had a high genetic diversity with VNI being the dominant genotype and these clinical strains also had low fluconazole MIC values. South African isolates with the molecular type VNB were not highly virulent in a *G. mellonella* larval model, but more research needs to be done to confirm this. The few South African VNB strains in our study were very closely related by WGS SNP analysis, suggesting that this genotype may be able to cause outbreaks. Two South African VNB strains were related to Brazilian VNB strains, suggesting some level of relatedness and donation of genetic information between strains across geographical regions. As we learn more about the relationships between molecular types of *C. neoformans* from different geographical locations, we will gain a better understanding of this fungus that is globally distributed throughout the world.

## Figures and Tables

**Table 1:** Fluconazole minimum inhibitory concentration (MIC) distribution (in µg/mL) by molecular type of 105 South African *Cryptococcus neoformans* isolates from national laboratory-based cryptococcosis surveillance collected between 2005 and 2009.

Molecular Type	Total	MIC Value										MIC_50_	MIC_90_	Geometric Mean	Range
		0.125	0.25	0.5	1	2	4	8	16	32	64				
VNI	60	0	2	10	20	19	3	5	0	1	0	1	6	1.43	0.25–32
VNB	15	0	1	2	6	3	3	0	0	0	0	1	4	1.26	0.25–4
VNII	25	0	0	5	15	4	1	0	0	0	0	1	2	1.03	0.5–4
VNIV	5	0	0	0	0	5	0	0	0	0	0	2	2	2	2
Total	105	0	3	17	41	31	7	5	0	1	0	1	4	1.32	0.25–32

**Table 2:** Virulence of *Cryptococcus neoformans* molecular type VNB strains inoculated into *Galleria mellonella* larvae compared to larvae inoculated with the highly-virulent H99 strain.

Strain	Median Survival Time	*p* Value
H99 *	2	NA
5873	2	0.54
205	3	0.02
2887	3	0.01
3438	3	0.24
1597	3	0.01
907	3	0.02
1239	3	0.04
3783	3	0.01
130	3	0.01
1543	3	0.01
637	4	0.02
1817	4	0.002
892	4	0.003
2890	4	0.001
1408	5	0.001

* H99 was used as the comparison strain in the log rank test of equality.

## Data Availability

The data presented in this study are available in the supplementary information for metadata.

## Supplementary Materials

The following are available online at https://www.mdpi.com/article/10.3390/jof7050338/s1, Spreadsheet: Supplementary information for metadata, Table S1: Characteristics of five South African patients infected with the *Cryptococcus neoformans* VNIV molecular type (serotype D) collected during national laboratory-based surveillance for cryptococcosis, 2005–2009, Table S2: Univariable logistic regression analysis to determine associations between clinical characteristics and infecting strain molecular type among South African patients infected with *Cryptococcus neoformans* serotype A (*n* = 246), 2005–2009, Table S3: Multivariable logistic regression analysis to determine associations between clinical characteristics and infecting strain molecular type among South African patients infected with *Cryptococcus neoformans* serotype A (*n* = 246), 2005–2009, Table S4: Univariable logistic regression analysis to determine associations between infecting strain molecular type and in-hospital outcome among South African patients (*n* = 180) infected with *Cryptococcus neoformans* serotype A, 2005–2009. Table S5: Multivariable logistic regression analysis to determine associations between infecting strain molecular type and in-hospital outcome among South African patients (*n* = 180) infected with *Cryptococcus neoformans* serotype A, 2005–2009, Table S6: Characteristics of 15 South African patients infected with the *Cryptococcus neoformans* VNB molecular type collected during national laboratory-based surveillance for cryptococcosis, 2005–2009.
