# Supplementary material for: Genotype, Antifungal Susceptibility, and Virulence of Clinical South African Cryptococcus neoformans Strains from National Surveillance, 2005–2009
Source: J Fungi (Basel). 2021 Apr 27;7(5):338. doi: 10.3390/jof7050338 (PMC8146981; doi:10.3390/jof7050338)
Supplement: Supplementary file 1 [file jof-07-00338-s001.zip › jof-1161502-supplementary/Table S1new submit.pdf]

Table S1: Characteristics of five South African patients infected with the *Cryptococcus neoformans* VNIV molecular type (serotype D) collected during national laboratory-based surveillance for cryptococcosis, 2005-2009

Isolate number	Home town/city	Province	Specimen collection date	Syndrome	Facility name
3562	Diepsloot	Gauteng	21/7/2006	Meningitis	Chris Hani Baragwanath
1168	Jeppestown	Gauteng	12/3/2007	Meningitis	Charlotte Maxeke Johannesburg Academic
1344	Unknown	Eastern Cape	22/2/2008	Unknown	Umtata General
3331	Cape Town	Western Cape	14/8/2008	Meningitis	Groote Schuur
3991	Jeppestown	Gauteng	26/11/2008	Unknown	Charlotte Maxeke Johannesburg Academic
