# Supplementary material for: Genotype, Antifungal Susceptibility, and Virulence of Clinical South African Cryptococcus neoformans Strains from National Surveillance, 2005–2009
Source: J Fungi (Basel). 2021 Apr 27;7(5):338. doi: 10.3390/jof7050338 (PMC8146981; doi:10.3390/jof7050338)
Supplement: Supplementary file 1 [file jof-07-00338-s001.zip › jof-1161502-supplementary/Table S2new submit.pdf]

Table S2: Univariable logistic regression analysis to determine associations between clinical characteristics and infecting strain molecular type among South African patients infected with *Cryptococcus neoformans* serotype A (*n* = 246), 2005-2009

Exposure variables	Non-VNI genotype<br>N=40	VNI genotype<br>N=206	Univariable analysis	
	n/N (%)	n/N (%)	OR (95% CI)	p-value
**Sex**				
Male	29/110 (26)	81/110 (74)	5.24 (2.28-12.04)	0.001
Female	8/125 (6)	117/125 (94)	reference	
Missing data (n)	3	8		
**Age (years)**				
<25	3/20 (15)	17/20 (85)	reference	
25-34	13/95 (14)	82/95 (86)	0.90 (0.23-3.50)	0.88
35-44	16/84 (19)	68/84 (81)	1.33 (0.35-5.11)	0.68
>45	5/34 (15)	29/34 (85)	0.98 (0.21-4.61)	0.98
Missing data (n)	3	10		
**Year of diagnosis**				
2005	7/50 (14)	43/50 (86)	reference	
2006	10/53 (19)	43/53 (81)	1.43 (0.50-4.10)	0.51
2007	7/52 (13)	45/52 (87)	0.96 (0.31-2.95)	0.94
2008	9/48 (19)	39/48 (81)	1.42 (0.48-4.17)	0.53
2009	7/43 (16)	36/43 (84)	1.19 (0.38-3.72)	0.76
**Province**				
Northern	18/123 (15)	105/123 (85)	0.81 (0.40-1.61)	0.54
Southern	20/114 (18)	94/114 (82)	reference	
Missing data (n)	2	7		
**Specimen type**				
Cerebrospinal fluid (CSF)	34/211 (16)	177/211 (84)	2.69 (0.34-21.14)	0.35
Blood	1/15 (7)	14/15 (93)	reference	
Missing data (n)	5	15		
**CD4+ T-cell count at diagnosis (cells/µl)**				
<=50	11/49 (22)	38/49 (78)	2.41 (0.61-9.52)	0.21
>50	3/28 (11)	25/28 (89)	reference	
Missing data (n)	26	143		
**Antiretroviral treatment**				
Yes	2/23 (9)	21/23 (91)	0.48 (0.10-2.23)	0.35
No	19/115 (17)	96/115 (83)	Reference	
Missing data (n)	19	89		
**Mental status at diagnosis***				
Alert	15/104 (14)	89/104 (86)	0.63 (0.27-1.49)	0.29
Not alert	11/52 (21)	41/52 (79)	reference	
Missing data (n)	14	76		
**CSF India ink result**				
Positive	27/164 (16)	137/164 (84)	0.39 (0.03-4.50)	0.45
Negative	1/3 (33)	2/3 (67)	reference	
Missing data (n)	12	67		
**CSF white cell count (cells/µl)**				
<5	7/56 (12.5)	49/56 (87.5)	0.58 (0.22-1.50)	0.26
>=5	17/86 (20)	69/86 (80)	reference	
Missing data (n)	16	88		
**Current antifungal treatment**				
Fluconazole alone	6/43 (14)	37/43 (86)	reference	
Fluconazole and amphotericin B	9/51 (18)	42/51 (82)	1.32 (0.43-4.06)	0.63
Missing data (n)	25	127		
**Current TB treatment**				
Yes	6/36 (17)	30/36 (83)	1.22 (0.42-3.49)	0.72
No	13/92 (14)	79/92 (86)	reference	
Missing data (n)	21	97		

*Mental status was categorised as “Alert” (Glasgow Coma Scale [GCS] score of 15) or “Not alert” (GCS score of <15 or recorded to be disorientated, stuporose or comatose).
