# Supplementary material for: Genotype, Antifungal Susceptibility, and Virulence of Clinical South African Cryptococcus neoformans Strains from National Surveillance, 2005–2009
Source: J Fungi (Basel). 2021 Apr 27;7(5):338. doi: 10.3390/jof7050338 (PMC8146981; doi:10.3390/jof7050338)
Supplement: Supplementary file 1 [file jof-07-00338-s001.zip › jof-1161502-supplementary/Table S3new submit.pdf]

Table S3: Multivariable logistic regression analysis to determine associations between clinical characteristics and infecting strain molecular type among South African patients infected with *Cryptococcus neoformans* serotype A (*n* = 246), 2005-2009

**Exposure variables**	**Non-VNI genotype N=40**	**VNI genotype N=206**	**Multivariable analysis**	
	**n/N (%)**	**n/N (%)**	**aOR (95% CI)**	**p-value**
**Sex**				
Male	29/110 (26)	81/110 (74)	1.65 (0.25-10.99)	0.61
Female	8/125 (6)	117/125 (94)	reference	
Missing data (n)	3	8		
**Age (years)**				
<25	3/20 (15)	17/20 (85)	reference	
25-34	13/95 (14)	82/95 (86)	0.62 (0.15-2.63)	0.52
35-44	16/84 (19)	68/84 (81)	0.84 (0.20-3.50)	0.81
>45	5/34 (15)	29/34 (85)	0.47 (0.09-2.47)	0.38
Missing data (n)	3	10		
**CD4+ T-cell count at diagnosis (cells/µl)**				
<=50	11/49 (22)	38/49 (78)	1.01 (0.13-7.95)	0.99
>50	3/28 (11)	25/28 (89)	reference	
Missing data (n)	26	143		
**Mental status at diagnosis***				
Alert	15/104 (14)	89/104 (86)	0.43 (0.06-2.96)	0.39
Not alert	11/52 (21)	41/52 (79)	reference	
Missing data (n)	14	76		
**CSF white cell count (cells/µl)**				
<5	7/56 (12.5)	49/56 (87.5)	0.65 (0.10-4.37)	0.66
>=5	17/86 (20)	69/86 (80)	reference	
Missing data (n)	16	88		

*Mental status was categorised as "Alert" (Glasgow Coma Scale [GCS] score of 15) or "Not alert" (GCS score of <15 or recorded to be disorientated, stuporose or comatose).
