# Supplementary material for: Genotype, Antifungal Susceptibility, and Virulence of Clinical South African Cryptococcus neoformans Strains from National Surveillance, 2005–2009
Source: J Fungi (Basel). 2021 Apr 27;7(5):338. doi: 10.3390/jof7050338 (PMC8146981; doi:10.3390/jof7050338)
Supplement: Supplementary file 1 [file jof-07-00338-s001.zip › jof-1161502-supplementary/Table S4new submit.pdf]

Table S4: Univariable logistic regression analysis to determine associations between infecting strain molecular type and in-hospital outcome among South African patients (*n* = 180) infected with *Cryptococcus neoformans* serotype A, 2005-2009

Exposure variables	Died N=64	Survived N=116	Univariable analysis	
	**n/N (%)**	**n/N (%)**	**OR (95% CI)**	**p-value**
**Molecular type**				
Non-VNI	9/31 (29)	22/31 (71)	0.69 (0.29-1.61)	0.39
VNI	54/145 (37)	91/145 (63)	reference	
Missing data (n)	1	3		
**Sex**				
Male	30/84 (36)	54/84 (64)	0.59 (0.24-1.47)	0.25
Female	33/95 (35)	62/95 (65)	reference	
Missing data (n)	1	0		
**Age (years)**				
<25	6/17 (35)	11/17 (65)	reference	
25-34	27/68 (40)	41/68 (60)	1.15 (0.38-3.49)	0.81
35-44	21/69 (30)	48/69 (70)	0.83 (0.27-2.54)	0.74
>45	10/26 (38)	16/26 (62)	1.33 (0.37-4.80)	0.67
**Year of diagnosis**				
2005	4/14 (29)	10/14 (71)	reference	
2006	15/42 (36)	27/42 (64)	1.47 (0.39-5.54)	0.57
2007	16/43 (37)	27/43 (63)	1.54 (0.41-5.76)	0.52
2008	12/43 (28)	31/43 (72)	0.93 (0.24-3.61)	0.92
2009	17/38 (45)	21/38 (55)	2.06 (0.55-7.77)	0.29
**Hospital**				
Academic	36/100 (36)	64/100 (64)	0.69 (0.30-1.60)	0.39
Regional	28/80 (35)	52/80 (65)	reference	
**Specimen type**				
Cerebrospinal fluid (CSF)	55/160 (34)	105/160 (66)	0.66 (0.27-1.62)	0.37
Blood	5/14 (36)	9/14 (64)	reference	
Missing data (n)	4	2		
**CD4+ T-cell count at diagnosis (cells/µl)**				
<=50	17/50 (34)	33/50 (66)	0.26 (0.05-1.42)	0.09
>50	8/28 (29)	20/28 (71)	reference	
Missing data (n)	39	63		
**Antiretroviral treatment**				
Yes	7/23 (30)	16/23 (70)	0.49 (0.15-1.58)	0.22
No	36/119 (30)	83/119 (70)	reference	
Missing data (n)	21	17		
**Mental status at diagnosis***				
Alert	29/105 (28)	76/105 (72)	0.63 (0.24-1.67)	0.35
Not alert	23/54 (43)	31/54 (57)	reference	
Missing data (n)	12	9		
**CSF white cell count (cells/µl)**				
<5	20/41 (49)	21/41 (51)	1.22 (0.42-3.52)	0.72
>=5	16/67 (24)	51/67 (76)	reference	
Missing data (n)	28	44		
**Current antifungal treatment**				
Fluconazole alone	11/45 (24)	34/45 (76)	reference	
Fluconazole and amphotericin B	13/52 (25)	39/52 (75)	1.02 (0.40-2.62)	0.96
Missing data (n)	40	43		
**TB treatment**				
Yes	15/37 (41)	22/37 (59)	0.73 (0.24-2.23)	0.58
No	26/95 (27)	69/95 (73)	reference	
Missing data (n)	23	25		

*Mental status was categorised as “Alert” (Glasgow Coma Scale [GCS] score of 15) or “Not alert” (GCS score of <15 or recorded to be disorientated, stuporose or comatose).
