# Supplementary material for: Genotype, Antifungal Susceptibility, and Virulence of Clinical South African Cryptococcus neoformans Strains from National Surveillance, 2005–2009
Source: J Fungi (Basel). 2021 Apr 27;7(5):338. doi: 10.3390/jof7050338 (PMC8146981; doi:10.3390/jof7050338)
Supplement: Supplementary file 1 [file jof-07-00338-s001.zip › jof-1161502-supplementary/Table S5new submit.pdf]

**Table S5**: Multivariable logistic regression analysis to determine associations between infecting strain molecular type and in-hospital outcome among South African patients (*n* = 180) infected with *Cryptococcus neoformans* serotype A, 2005-2009

| **Exposure variables** | **Died N=64** | **Survived N=116** | **Multivariable analysis** | |
|---|---|---|---|---|
| | **n/N (%)** | **n/N (%)** | **aOR (95% CI)** | **p-value** |
| **Molecular type** | | | | |
| Non-VNI | 9/31 (29) | 22/31 (71) | 0.50 (0.03-7.57) | 0.62 |
| VNI | 54/145 (37) | 91/145 (63) | reference | |
| Missing data (n) | 1 | 3 | | |
| **Sex** | | | | |
| Male | 30/84 (36) | 54/84 (64) | 0.56 (0.06-5.60) | 0.62 |
| Female | 33/95 (35) | 62/95 (65) | reference | |
| Missing data (n) | 1 | 0 | | |
| **Age (years)** | | | | |
| <25 | 6/17 (35) | 11/17 (65) | reference | |
| 25-34 | 27/68 (40) | 41/68 (60) | 0.25 (0.01-9.87) | 0.46 |
| 35-44 | 21/69 (30) | 48/69 (70) | 0.35 (0.01-14.84) | 0.58 |
| >45 | 10/26 (38) | 16/26 (62) | 0.55 (0.01-46.60) | 0.79 |
| **CD4+ T-cell count at diagnosis (cells/µl)** | | | | |
| <=50 | 17/50 (34) | 33/50 (66) | 1.15 (0.13-10.29) | 0.90 |
| >50 | 8/28 (29) | 20/28 (71) | reference | |
| Missing data (n) | 39 | 63 | | |
| **Antiretroviral treatment** | | | | |
| Yes | 7/23 (30) | 16/23 (70) | 20.20 (0.88-464.75) | 0.06 |
| No | 36/119 (30) | 83/119 (70) | reference | |
| Missing data (n) | 21 | 17 | | |
| **Mental status at diagnosis*** | | | | |
| Alert | 29/105 (28) | 76/105 (72) | 0.16 (0.01-2.10) | 0.16 |
| Not alert | 23/54 (43) | 31/54 (57) | reference | |
| Missing data (n) | 12 | 9 | | |
| **Current antifungal treatment** | | | | |
| Fluconazole alone | 11/45 (24) | 34/45 (76) | reference | |
| Fluconazole and amphotericin B | 13/52 (25) | 39/52 (75) | 2.56 (0.22-30.21) | 0.46 |
| Missing data (n) | 40 | 43 | | |

*Mental status was categorised as "Alert" (Glasgow Coma Scale [GCS] score of 15) or "Not alert" (GCS score of <15 or recorded to be disorientated, stuporose or comatose).
