# Supplementary material for: Genotype, Antifungal Susceptibility, and Virulence of Clinical South African Cryptococcus neoformans Strains from National Surveillance, 2005–2009
Source: J Fungi (Basel). 2021 Apr 27;7(5):338. doi: 10.3390/jof7050338 (PMC8146981; doi:10.3390/jof7050338)
Supplement: Supplementary file 1 [file jof-07-00338-s001.zip › jof-1161502-supplementary/Table S6new submit.pdf]

Table S6: Characteristics of 15 South African patients infected with the *Cryptococcus neoformans* VNB molecular type collected during national laboratory-based surveillance for cryptococcosis, 2005-2009

**Isolate number**	**Home town/city**	**Province**	**Specimen collection date**	**Syndrome**	**Facility name**
205	Meadowlands	Gauteng	26/1/2005	Meningitis	Chris Hani Baragwanath
892	Soweto	Gauteng	25/2/2006	Meningitis	Chris Hani Baragwanath
1817	Mangaung	Free State	18/4/2006	Meningitis	Pelonomi
130	Mthatha	Eastern Cape	9/1/2009	Meningitis	Nelson Mandela Academic/Mthatha Provincial
2887	Kroonstad	Free State	29/8/2005	Unknown	Boitumelo
3783	Johannesburg	Gauteng	26/10/2008	Meningitis	Chris Hani Baragwanath
5873	Soweto	Gauteng	8/11/2006	Meningitis and pneumonia	Chris Hani Baragwanath
1239	Orange Farm	Gauteng	15/3/2007	Meningitis	Chris Hani Baragwanath
907	Soweto	Gauteng	26/2/2006	Meningitis	Chris Hani Baragwanath
3438	Unknown	North West	9/10/2005	Unknown	Bophelong/Mafikeng
1597	Lenasia	Gauteng	2/4/2006	Meningitis	Chris Hani Baragwanath
1543	Tshwane	Gauteng	16/7/2009	Meningitis	Steve Biko Pretoria Academic
1408	Boksburg	Gauteng	19/4/2005	Unknown	Tambo Memorial
637	Scottburgh	KwaZulu-Natal	9/2/2005	Unknown	GJ Crooke's/Scottburgh
2890	Rouxville	Free State	26/6/2008	Pneumonia	Pelonomi
